# Establishment of regulatory elements during erythro-megakaryopoiesis identifies hematopoietic lineage-commitment points

**DOI:** 10.1186/s13072-018-0195-z

**Published:** 2018-05-28

**Authors:** Elisabeth F. Heuston, Cheryl A. Keller, Jens Lichtenberg, Belinda Giardine, Stacie M. Anderson, NIH Intramural Sequencing Center, Ross C. Hardison, David M. Bodine

**Affiliations:** 1 NHGRI Hematopoiesis Section, GMBB, Bethesda, MD USA; 2 Pennsylvania State University, University Park, PA USA; 3 NHGRI Flow Cytometry Core, Bethesda, MD USA; 4 NHGRI, Rockville, MD USA

**Keywords:** Hematopoiesis, Megakaryopoiesis, Erythropoiesis, ATAC-Seq, RNA-Seq, ChIP-Seq, Lineage commitment, Epigenetics

## Abstract

**Background:**

Enhancers and promoters are cis-acting regulatory elements associated with lineage-specific gene expression. Previous studies showed that different categories of active regulatory elements are in regions of open chromatin, and each category is associated with a specific subset of post-translationally marked histones. These regulatory elements are systematically activated and repressed to promote commitment of hematopoietic stem cells along separate differentiation paths, including the closely related erythrocyte (ERY) and megakaryocyte (MK) lineages. However, the order in which these decisions are made remains unclear.

**Results:**

To characterize the order of cell fate decisions during hematopoiesis, we collected primary cells from mouse bone marrow and isolated 10 hematopoietic populations to generate transcriptomes and genome-wide maps of chromatin accessibility and histone H3 acetylated at lysine 27 binding (H3K27ac). Principle component analysis of transcriptional and open chromatin profiles demonstrated that cells of the megakaryocyte lineage group closely with multipotent progenitor populations, whereas erythroid cells form a separate group distinct from other populations. Using H3K27ac and open chromatin profiles, we showed that 89% of immature MK (iMK)-specific active regulatory regions are present in the most primitive hematopoietic cells, 46% of which contain active enhancer marks. These candidate active enhancers are enriched for transcription factor binding site motifs for megakaryopoiesis-essential proteins, including ERG and ETS1. In comparison, only 64% of ERY-specific active regulatory regions are present in the most primitive hematopoietic cells, 20% of which containing active enhancer marks. These regions were not enriched for any transcription factor consensus sequences. Incorporation of genome-wide DNA methylation identified significant levels of de novo methylation in iMK, but not ERY.

**Conclusions:**

Our results demonstrate that megakaryopoietic profiles are established early in hematopoiesis and are present in the majority of the hematopoietic progenitor population. However, megakaryopoiesis does not constitute a “default” differentiation pathway, as extensive de novo DNA methylation accompanies megakaryopoietic commitment. In contrast, erythropoietic profiles are not established until a later stage of hematopoiesis, and require more dramatic changes to the transcriptional and epigenetic programs. These data provide important insights into lineage commitment and can contribute to ongoing studies related to diseases associated with differentiation defects.

**Electronic supplementary material:**

The online version of this article (10.1186/s13072-018-0195-z) contains supplementary material, which is available to authorized users.

## Background

Hematopoiesis is the process by which proliferating hematopoietic stem cells (HSC) undergo continued transcriptional and epigenetic changes associated with lineage-restriction and cell-specific function, giving rise to all of the cell types in the hematopoietic system. Classical models of hematopoiesis describe a series of progressively restricted cell fate decisions in which HSC give rise to multipotent progenitors (MPP), which in turn give rise to common myeloid progenitors (CMP) and common lymphoid progenitors (CLP). In myelopoiesis, CMP differentiate into granulocyte–macrophage progenitors (GMP), from which mature granulocytes and monocytes are generated, and megakaryocytic erythroid progenitors (MEP), which have classically been described as the common erythrocyte and megakaryocyte progenitor [1–4].

**Fig. 1:**
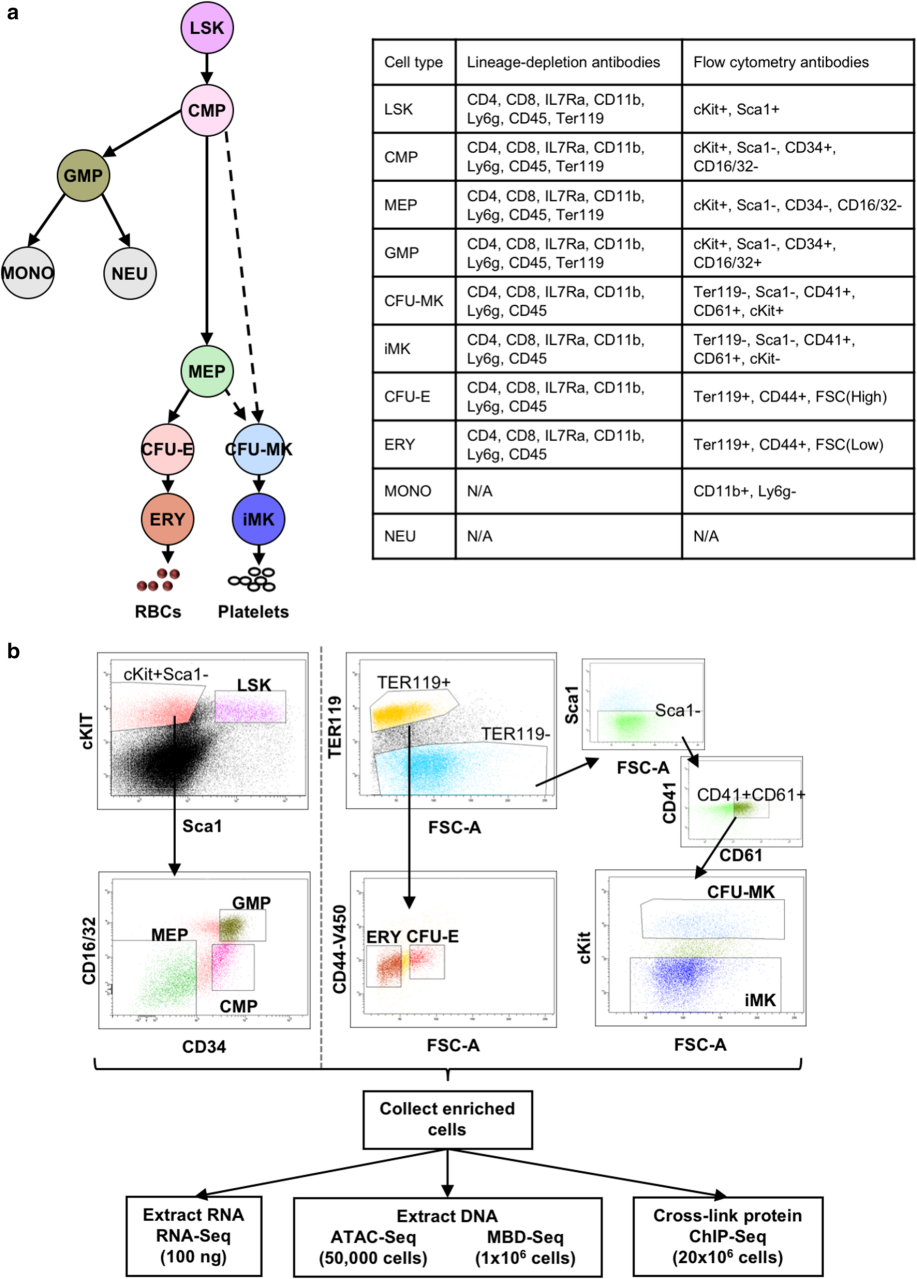
Primary mouse bone marrow cells isolated via flow cytometry. **a** Hematopoietic populations assayed in this study. Branches represent lineage-commitment points. Cell surface markers used for flow cytometry enrichment are shown. **b** Sort logic is shown for flow cytometry enrichment

Erythro-megakaryopoiesis describes the subset of cell fate decisions associated with the production of erythroblasts (ERY; erythropoiesis) and megakaryocytes (MK; megakaryopoiesis). The process of erythroid and megakaryocyte commitment is accompanied by the substitution of GATA1 for GATA2 at key chromatin regulatory sites to increase expression of linage-specific transcription factors including FLI1 and ETS in the MK lineage, and KLF1 and GATA1 in the ERY lineage [5–8]. Traditional hierarchical models predict that ERY and MK lineages are derived from a homogeneous population of bipotential MEP (Fig. 1a) [9]. However, there is mounting evidence that the MK program is established prior to the emergence of erythroid cells. Several studies have noted transcriptional and immunophenotypic similarities between HSC and MK populations [10–13]. Single-cell transcriptional studies of human MEP demonstrated that traditionally defined MEP included erythroid- and megakaryocyte-committed cells mixed with a small compartment of bipotential intermediates [14]. These studies have led to revised models where many MK are derived from a multipotent stem cell, while others can be generated from CMP and MEP intermediates [15]. While MK appears to have multiple origins, in these revised models the erythroid lineage appears to remain a single branch downstream of the CMP and formed through the MEP.

Determining when lineage specification occurs is important for understanding mechanisms behind diseases with maturation defects. These diseases include anemias, thrombocytopenias, neutropenias, myelodysplastic and myeloproliferative disorders, and hematologic malignancies [16–20]. Fluorescence-activated cell sorting and next-generation sequencing technologies make it possible to generate transcriptome profiles (RNA-Seq), genome-wide profiles of chromatin accessibility (ATAC-Seq), histone modifications (ChIP-Seq), and DNA methylation (MBD-Seq) of cells at specific stages of hematopoiesis. As part of the ValIdated Systematic IntegratiON of hematopoietic epigenomes (VISION) project, we are creating comprehensive catalogs of genomic regulatory elements in primary mouse cells to compare and contrast epigenetic regulation in mice with human data from the Encyclopedia of DNA Elements (ENCODE) project and the NIH Roadmap Epigenomics Project [21–24]. VISION also emphasizes bioinformatics modeling and machine learning to predict regulatory interactions which is enabled by the ability to manipulate mouse hematopoiesis at the genetic level.

To characterize the relationships between hematopoietic populations and the changes that accompany erythro-megakaryopoiesis, we enriched 10 primary hematopoietic cell populations from C57BL6 mice for RNA-Seq, ATAC-Seq, ChIP-Seq, and MBD-Seq analysis. These data have been integrated into transcriptional and epigenetic maps to identify candidate regulatory elements in the ERY and MK lineages and to study the establishment and maintenance of these regulatory elements during erythro-megakaryopoiesis. Our results demonstrate that committed megakaryocytes have similar transcriptional and epigenetic profiles to hematopoietic stem and progenitor cells, but do not represent a “default” developmental program. By contrast, the erythroid population is the most dissimilar from hematopoietic stem and progenitor populations and requires more extensive changes to the transcriptional and epigenetic programs to permit erythropoiesis.

## Results

### Committed megakaryocyte precursors have similar transcriptional profiles to hematopoietic progenitor cells

RNA-Seq was performed on two biological replicates of total RNA isolated from 10 populations of primary mouse bone marrow separated on the basis of cell surface marker expression (Fig. 1a, b). We defined “expressed” transcripts as having a transcripts per million count (TPM) ≥ 1 (Table 1). Because our primary megakaryocytes were not treated with thrombopoietin, these cells do not express many of the genes involved in platelet function; we therefore refer to this population as immature MK (iMK).

**Table 1:** Numbers of features ascertained in each cell population

Population	Number of transcripts (TPM ≥ 1)	Number of transcripts (TPM ≥ 10)	Number of ATAC peaks	Number of methylation peaks
LSK	13,066	7515	64,043	33,754
CMP	12,348	7065	107,281	10,288
CFU-E	9631	3834	41,805	16,439
ERY	10,025	3525	42,260	5218
CFU-MK	11,889	7059	122,757	69,405
iMK	11,751	6848	97,463	52,754
MEP	11,574	6834	76,334	N/A
GMP	11,749	6972	112,947	N/A
NEU	12,250	6330	44,239	N/A
MONO	11,857	6239	53,531	N/A

**Fig. 2:**
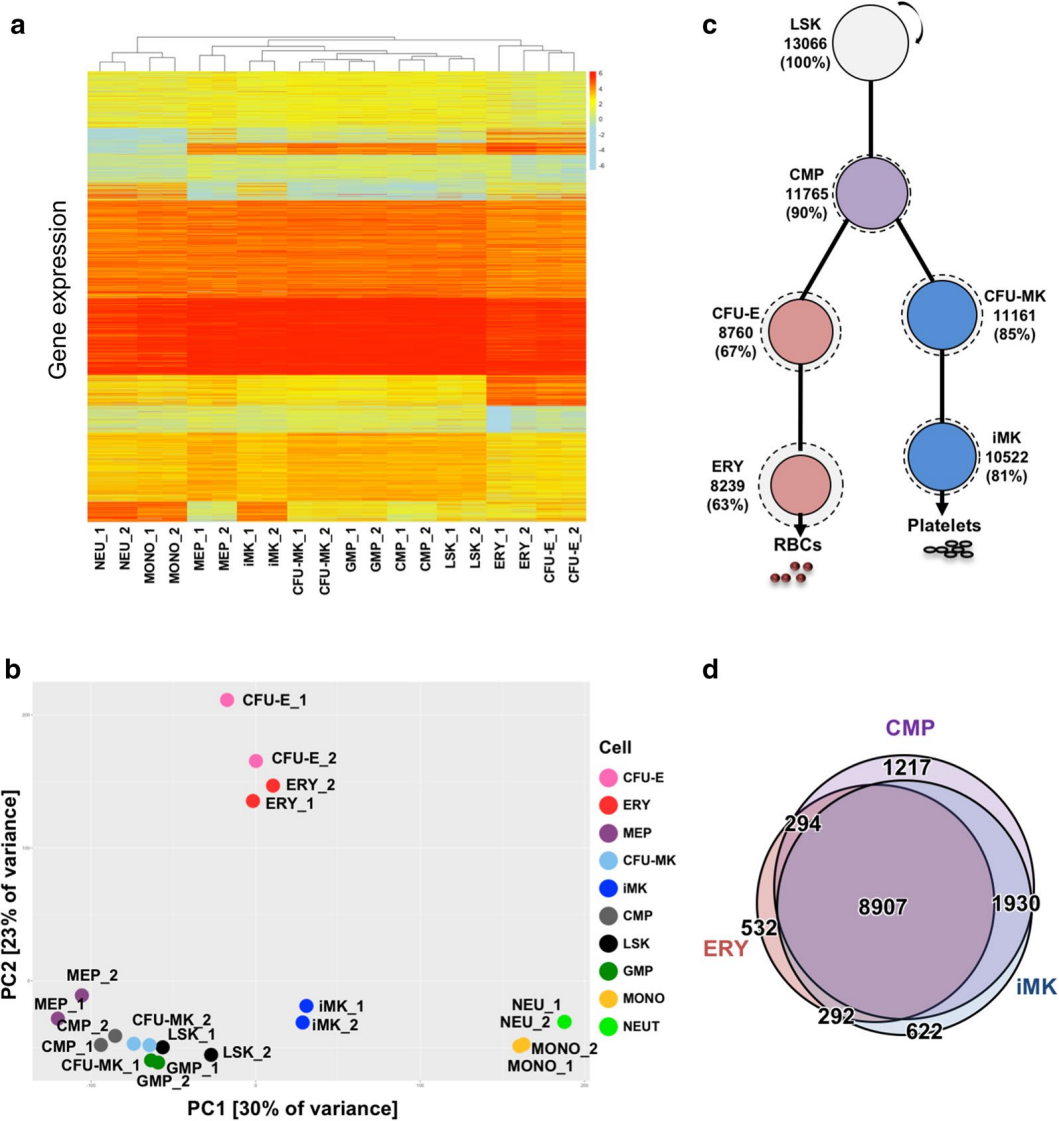
Transcriptional profiles of hematopoietic populations. **a** Unsupervised hierarchical clustering analysis of RNA-Seq-derived transcriptomes. **b** Principle component analysis plotting PC1 versus PC2. **c** Expressed transcripts in LSK and maintained throughout erythro-megakaryopoiesis. Solid circle size is proportional to the percentage of expressed transcripts maintained from the LSK, dotted circle is proportional to the percentage of all transcripts in the cell relative to the number maintained from LSK, and line length is inversely proportional to percentage of expressed transcripts maintained from the progenitor. The number and percentage of maintained LSK transcripts is shown. **d** Proportional Venn diagram indicating overlap of expressed genes between CMP, ERY, and iMK populations

Unsupervised hierarchical clustering of the individual RNA-Seq replicates showed that lineage-restricted megakaryocytic populations (CFU-MK and iMK) clustered with progenitors (LSK, CMP, GMP, and MEP), while lineage-restricted erythroid (CFU-E and ERY) and granulocyte populations grouped in different clusters (Fig. 2a). We used principal component analysis (PCA) to display relationships among the transcriptomes on a plane representing the largest variance in the datasets. As expected, replicates for each cell population fell closer to each other than to other cell types. Principal component 1 (PC1), which accounted for 30% of the variance, separated erythroid, monocyte, and neutrophil cells from progenitor cells. PC2, which accounted for 23% of the total variance, separated erythroid cells from all other cells (Fig. 2b). Strikingly, the transcriptome of the committed MK progenitor, CFU-MK, grouped with progenitor cells, while the iMK grouped closest to this population.

Of the 13,000 transcripts expressed in our multipotent progenitor population (LSK), 81% were also expressed in the iMK population (Fig. 2c, solid circle); this fraction represents 89% of all iMK transcripts (Fig. 2c, dotted circle). In contrast, 63% of transcripts expressed in LSK were expressed in ERY, representing 82% of all ERY transcripts. This fraction is significantly smaller than that observed for the iMK population (proportion test *p* value < 0.001) (Fig. 2c, Table 2). Of the almost 12,000 expressed CMP transcripts, 1930 (16%) were expressed in iMK but not in ERY, whereas only 294 (2%) were expressed in ERY but not in iMK (proportion test *p* value < 0.001) (Fig. 2d, Table 2). These results show that cellular transcriptomes of the megakaryocytic lineage are similar to those of multilineage progenitor cells, whereas erythroid cells repress the multilineage transcriptome.

**Table 2:** Number of transcripts with maintained expression at subsequent stages of hematopoiesis

	Expressed in LSK and CMP	Expressed in LSK, CMP, and committed progenitor	Expressed in LSK, CMP, committed progenitor, and mature cell
ERY			
# Expressed since LSK	11,765	8760	8239
% Expressed since LSK	90	67	63
% Expressed from immediate progenitor	90	74	94
iMK			
# Expressed since LSK	11,765	11,161	10,522
% Expressed since LSK	90	85	81
% Expressed from immediate progenitor	90	95	94

### Committed megakaryocytes have a similar chromatin accessibility profile to hematopoietic progenitor cells

**Fig. 3:**
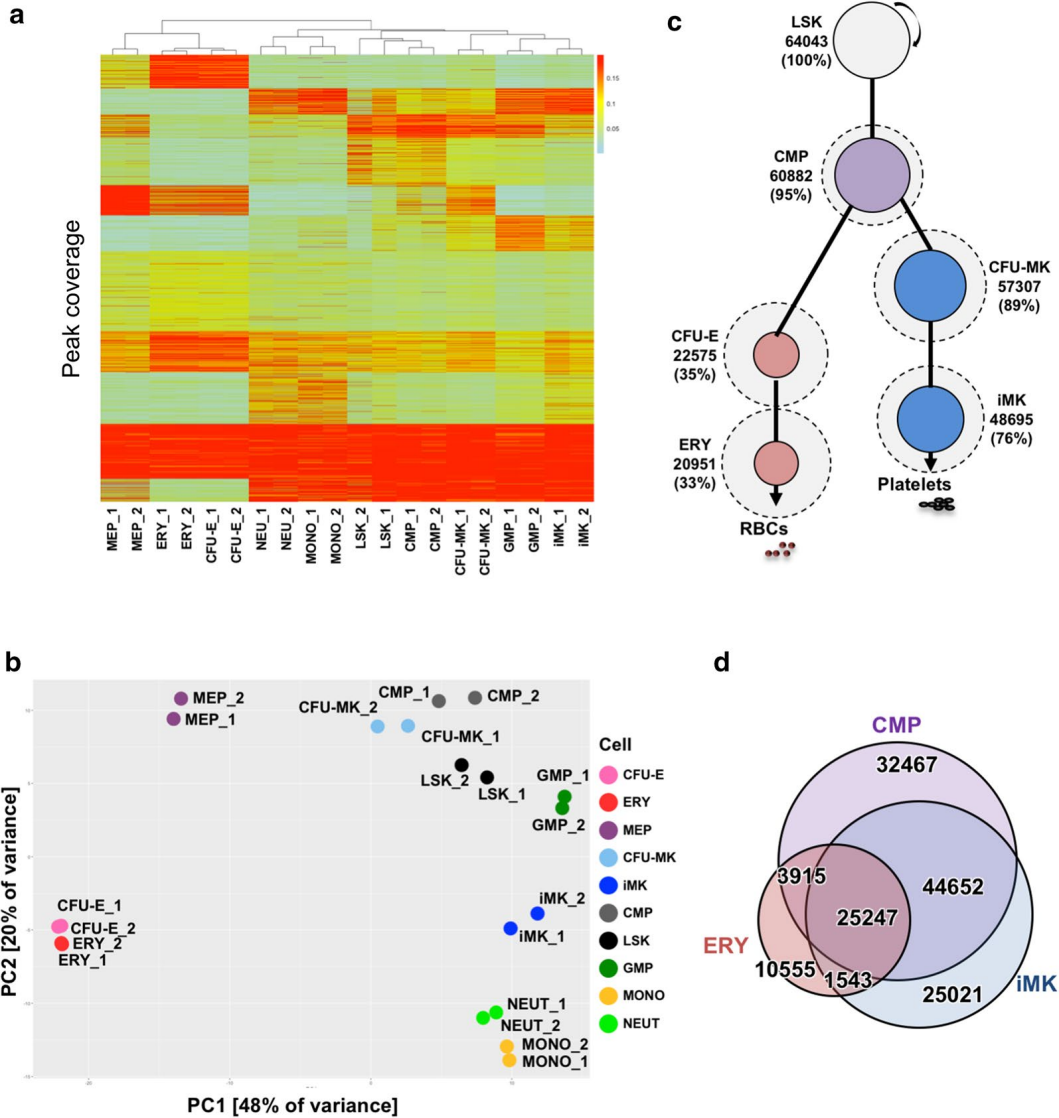
ATAC-Seq profiles of hematopoietic populations. **a** Unsupervised hierarchical clustering analysis of ATAC-Seq-derived peak profiles. **b** Principle component analysis plotting PC1 versus. **c** ATAC-Seq peaks in LSK and maintained throughout erythro-megakaryopoiesis. Circle size is proportional to the percentage of peaks maintained from the LSK, dotted circle is proportional to the percentage of all peaks in the cell relative to the number maintained from LSK, and line length is inversely proportional to percentage of peaks maintained from the progenitor. The number and percentage of maintained LSK peaks is shown. **d** Proportional Venn diagram indicating overlap of ATAC-Seq peaks between CMP, ERY, and iMK populations

We generated maps of accessible chromatin regions using the Assay for Transposase-Accessible Chromatin (ATAC) on the same populations of primary mouse bone marrow cells used for transcriptional profiling. Unsupervised hierarchical clustering of the average ATAC-Seq signal in each peak region showed that, like the transcriptional profiles, the ATAC profiles of iMK and CFU-MK clustered with LSK, CMP, and GMP, while the ATAC profiles of the erythroid, neutrophil, and monocyte populations clustered separately (Fig. 3a). These groups of committed erythroid cells and committed granulocytes were also observed in the PCA, while CFU-MK and iMK again grouped with the multilineage progenitor cells (Fig. 3b). Unlike the transcriptional profiles, profiles of MEP chromatin accessibility clustered with erythroid cells rather than with the multilineage progenitors.

Of ~ 64,000 ATAC-Seq peaks in LSK, more than 95% of these were present in the set of ~ 100,000 CMP peaks (Fig. 3c, Table 1). Similarly, 76% (~ 48,695) of LSK ATAC-Seq peaks were present in the set of ~ 97,000 iMK peaks (representing 72% of all iMK peaks) (Fig. 3c, Table 1). In contrast, only 33% (~ 21,000) of LSK ATAC-Seq peaks were present in the set of ~ 42,000 ERY peaks (representing 50% of all ERY peaks). While less overlap was observed among CMP, ERY, and iMK ATAC-Seq profiles compared to the overlap observed in RNA-Seq profiles, a substantially larger number of peaks were shared between iMK and CMP than between ERY and CMP (Fig. 3d). We conclude that iMK maintains much of the transposase-accessible chromatin that is established in LSK, while erythropoiesis involves significant loss of accessibility in chromatin that was open in progenitors, implying substantial compaction of chromatin during erythropoiesis.

### Megakaryocytic regulatory elements are established in multipotent progenitor cells, while many erythroid regulatory elements are established in more differentiated cells

**Fig. 4:**
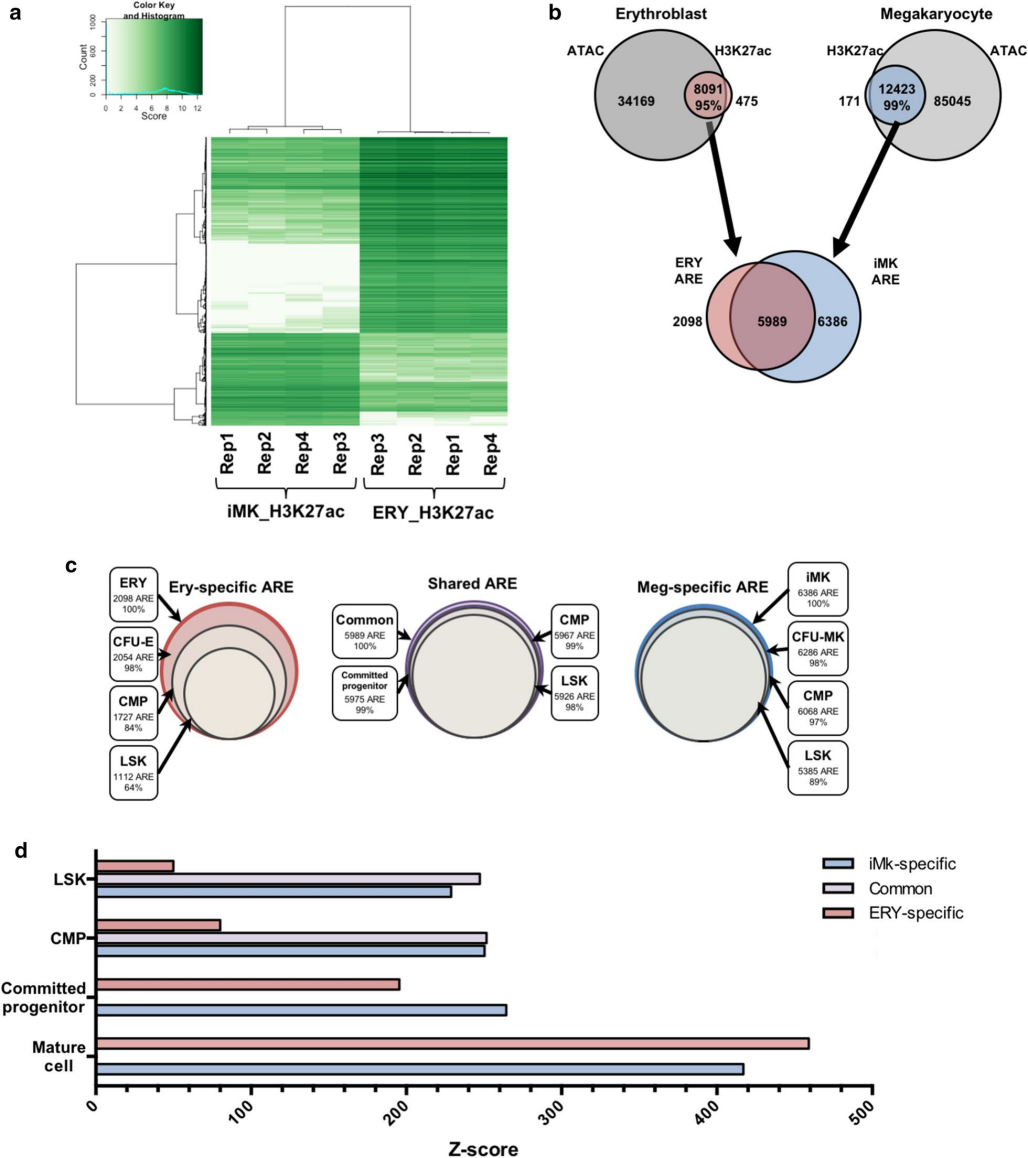
Establishment of ERY and iMK AREs throughout hematopoiesis. **a** Heatmap comparing H3K27ac immunoprecipitation peaks in ERY and iMK samples. Calculations were performed using the R package DiffBind (v2.2.6). **b** Active regions are defined as the intersection of ATAC and H3K27ac peaks in ERY and iMK. **c** Establishment of open chromatin was defined as intersecting AREs from ERR or iMK with ATAC-Seq peaks in sequentially more primitive cell populations. **d** Significance of overlap was calculated by randomizing peak positions and calculating random versus expected overlap (500 iterations). *Z*-score of overlap between AREs and ATAC peaks. Hashed bars indicate *p* value > 0.05

Active enhancers and promoters are marked by the presence of histone H3 acetylated at lysine 27 (H3K27ac) [25]. We generated genome-wide maps of candidate enhancers and promoters in ERY and iMK by performing ChIP-Seq of H3K27ac. Using four replicates for each population, we identified 8566 ERY and 12,594 iMK H3K27ac peaks (Fig. 4a, b). Since active enhancers and promoters are almost always in regions of open chromatin, we generated a set of high-confidence active regulatory elements (AREs) by overlapping the H3K27ac peaks with ATAC-Seq peaks. We found that over 95% of H3K27ac peaks overlapped with ATAC regions. We identified 2098 ERY-specific and 6386 iMK-specific AREs, and 5989 common AREs shared by both cell types (Fig. 4b). We assigned AREs to candidate target genes using the closest transcriptional start site (TSS), regardless of distance or activation state. This method assigned the majority of AREs to actively expressed candidate target genes: 82% of ERY-specific AREs, 89% of iMK-specific AREs, and 93% of shared AREs (Table 3). To infer the stage at which ERY or iMK AREs appear (inferred AREs), we intersected the sets of ERY and iMK AREs with the ATAC-Seq peaks of progenitor cells (Fig. 4c). Between 80 and 90% of these inferred AREs were associated with an actively transcribed gene (Table 3). Over 98% of shared ERY and iMK AREs were present in LSK. 89% of iMK-specific AREs were present in LSK, while 97% were present in CMP. To calculate the significance of the overlap, we performed permutation tests with the R package “regioneR” [26]. This analysis demonstrated that the observed number of overlaps was statistically higher than one would expect by random chance (LSK *p* value 0.002, CMP *p* value 0.002) (Fig. 4d). In contrast, while 64% of ERY-specific AREs had already been established in LSK, this portion is significantly lower compared to 89% of iMK-specific AREs (*p* value 0.002). More ERY-specific AREs (84%) were present in CMP, but this was significantly lower than the 97% of iMK-specific AREs (*p* value 0.002). Approximately 14% of ERY-specific AREs were established de novo in CFU-E (*p* value 0.002), whereas only 1% of iMK-specific AREs are established de novo in CFU-MK (*p* value 0.002) (Fig. 4d). We conclude that the regulatory element profiles showed a greater amount of lineage-specific activation in ERY than in iMK, similar to what was observed for the transcriptional profiles.

**Table 3:** Number of AREs assigned to the closest TSS

ARE establishment stage	Category	ERY-specific		Common		MEG-specific	
LSK	AREs	1112		5926		5385	
	No. genes assigned as ARE targets	1118		7963		4462	
	No. genes expressed	940	84%	7459	93%	3965	89%
CMP	AREs	1727		5967		6068	
	No. genes assigned as ARE targets	1606		7982		4767	
	No. genes expressed	1294	81%	7415	94%	4083	86%
Committed progenitor	AREs	2054		5975		6286	
	No. genes assigned as ARE targets	1829		7987		4848	
	No. genes expressed	1443	79%	7266	90%	4238	87%
Terminally differentiated cell	AREs	2098		5989		6386	
	No. genes assigned as ARE targets	1853		7998		4890	
	No. genes expressed	1527	82%	7464	93%	4341	89%

**Fig. 5:**
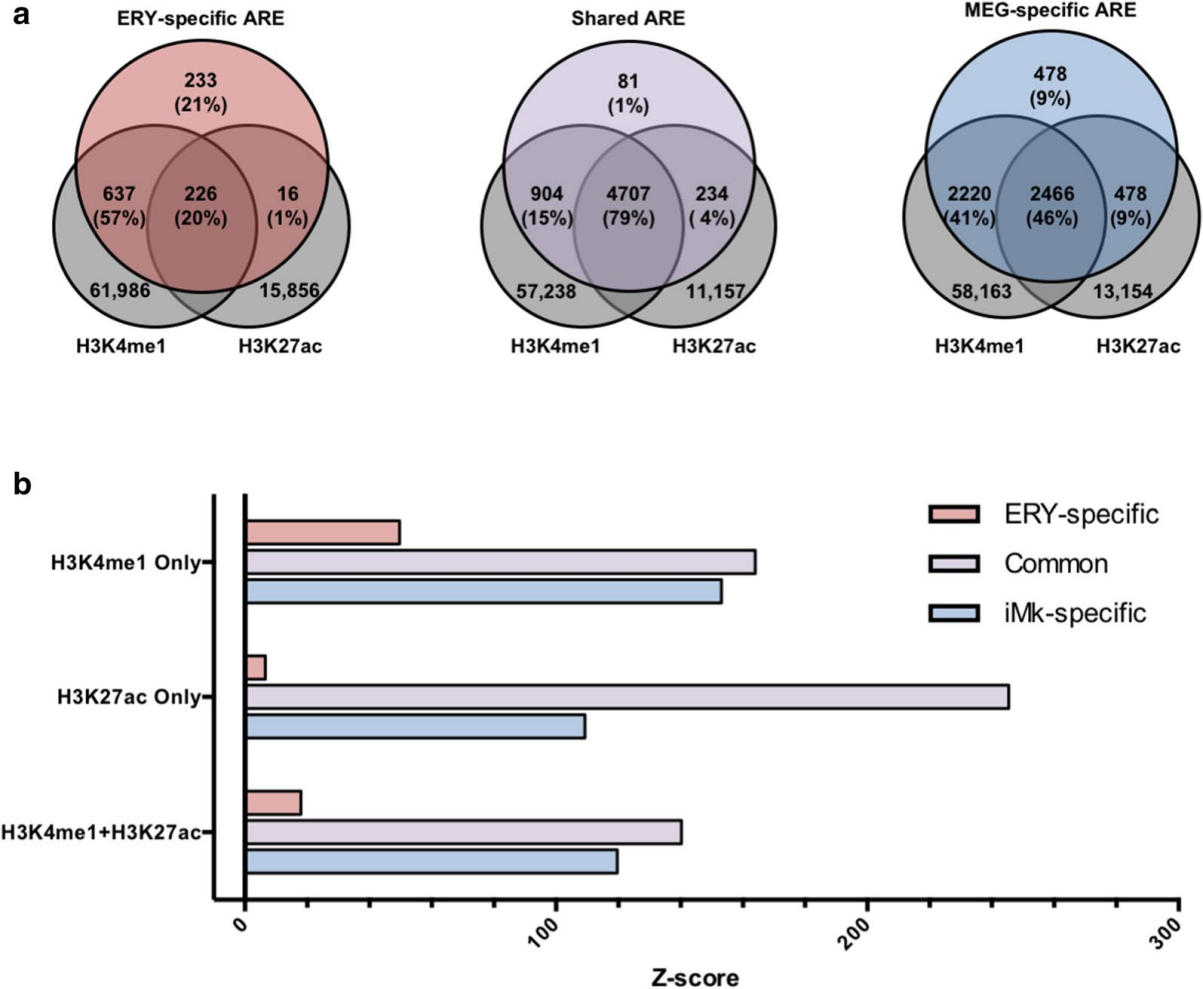
Establishment of active and poised enhancers in LSK. **a** LSK-accessible cell-specific and shared AREs were compared against the indexing-first H3K4me1 and H3K27ac chromatin immunoprecipitation profiles (Lara-Astiaso et al, Science, 2014). **b** *Z*-score of overlap between ARE and iChIP peaks

Poised enhancers are defined as regions of accessible chromatin that contain histone H3 monomethylated at lysine 4 (H3K4me1), but they can be distinguished from active enhancers by the absence of H3K27 acetylation [25]. To determine whether the inferred AREs in progenitor cells were poised (H3K4me1 only) or active (H3K4me1 and H3K27ac) in LSK, we compared our data with the indexing-first (iChIP) H3K4me1 and H3K27ac regions identified by Lara-Astiaso et al. [27]. We accessed iChIP sequencing reads from Gene Omnibus (GSE60103) and performed genome alignment and peak calling using our pipeline (see Materials and Methods). This analysis identified 62,849 H3K4me1 and 16,098 H3K27ac peaks in the iChIP LSK set. Approximately 57% of the LSK-established ERY-specific and 41% iMK-specific AREs overlapped with H3K4me1 peaks but not H3K27ac peaks, classifying them as poised enhancers (Fig. 5a, Additional file 1: Fig. S1). Approximately 20% of LSK-established ERY-specific and 46% of LSK-established iMK-specific AREs overlapped with both H3K4me1 and H3K27ac, classifying them as active enhancers. In comparison, 15 and 79% of LSK-accessible shared AREs were classified as poised and active enhancers, respectively. 21% of LSK-established ERY-specific and 9% of LSK-established iMK-specific AREs did not overlap with either H3K4me1 or H3K27ac peaks, and were therefore classified as open AREs. As validation, 23 of our ERY AREs overlapped with a set of 39 enhancers validated by luciferase assays [28], while none were found in the set of open AREs. Permutations tests using 500 iterations of randomized peak overlaps demonstrated that these intersections were statistically higher than expected by chance alone (Fig. 5b).

**Fig. 6:**
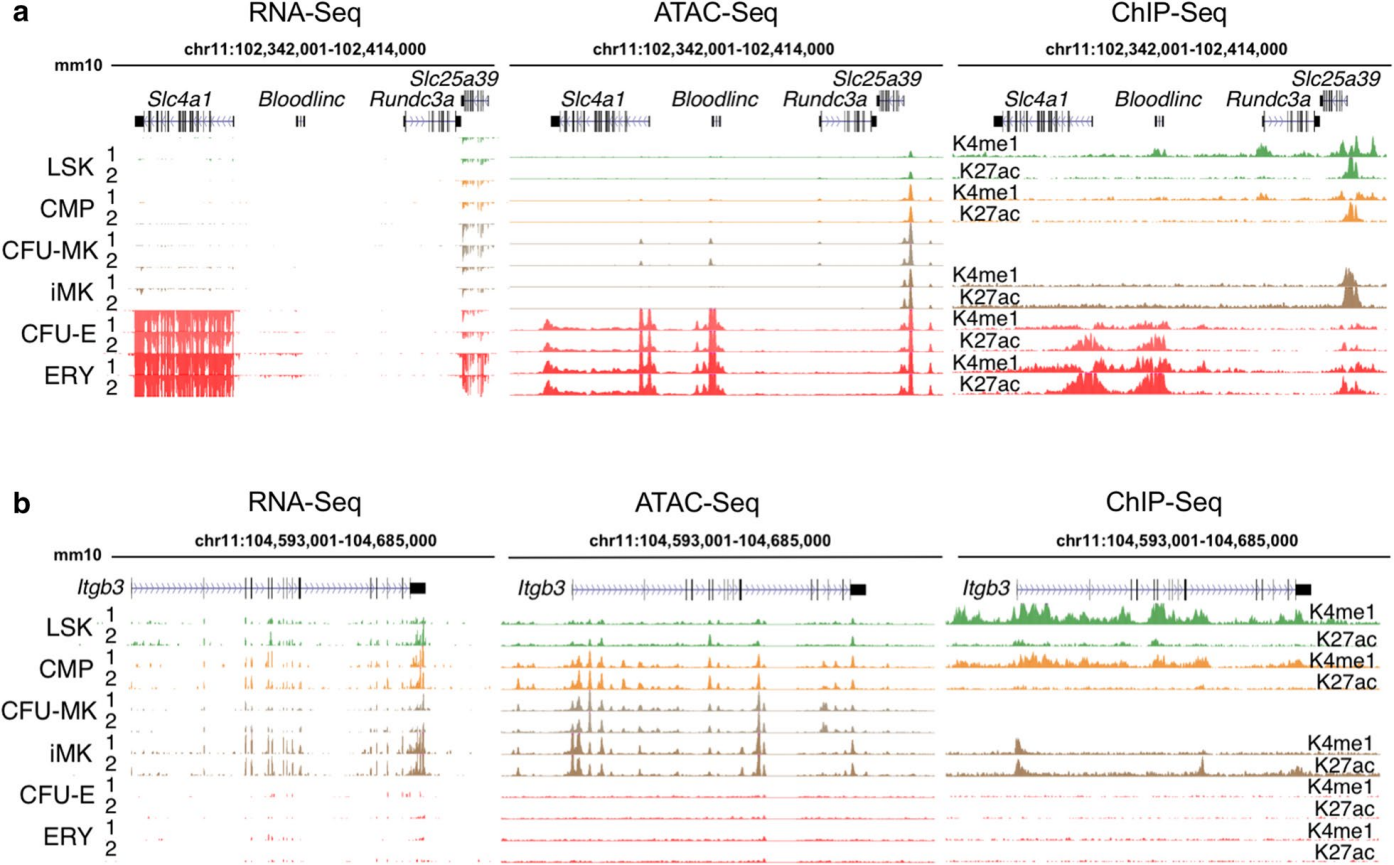
Transcriptional and epigenetic features illustrating different modes of regulation. **a** Induction of expression and AREs at *Slc4a1, Bloodlinc,* and *Slc25a39*. RNA-Seq data (left panel) are shown for each pair of replicates only for transcription of the minus strand (and thus RNA from *Rundc3a* is not shown). ATAC-Seq patterns (central panel) are shown for each pair of replicates. Histone modifications (right panel) are shown as single determinations, although some are available as replicates. **b** Retention of expression and AREs and from LSKs to MKs with loss in ERY for *Itgb3*. Displays are arranged as in (**a**) except RNA-Seq is shown for the plus strand

To visualize the integration of RNA-Seq, ATAC-Seq, and ChIP-Seq profiles, we examined several genes significant for ERY and iMK maturation (Fig. 6, Additional file 2: Fig. S2). The gene *Slc4a1*, encoding the anion transporter BAND3, is strongly induced during erythroid maturation. This induction was accompanied by the ERY-specific induction of transposase-accessible chromatin around the promoter and 3′ end of the gene, as well as in the upstream noncoding gene *Bloodlinc* (Fig. 6a). Chromatin in these regions was also was modified with H3K27ac and H3K4me1, indicative of active elements (Fig. 6a). The closely linked gene *Slc25a39*, encoding a constitutively expressed mitochondrial carrier protein, was in accessible chromatin in all the examined cell types (Fig. 6a). In contrast, the *Itgb3* gene (encoding the surface marker glycoprotein IIIa or CD61) was expressed and in regions of open chromatin in LSK, CMP, and MK-committed populations, but was repressed and in non-accessible chromatin regions in erythroid cells (Fig. 6b).

### Differing properties of ERY- and iMK-specific AREs

**Fig. 7:**
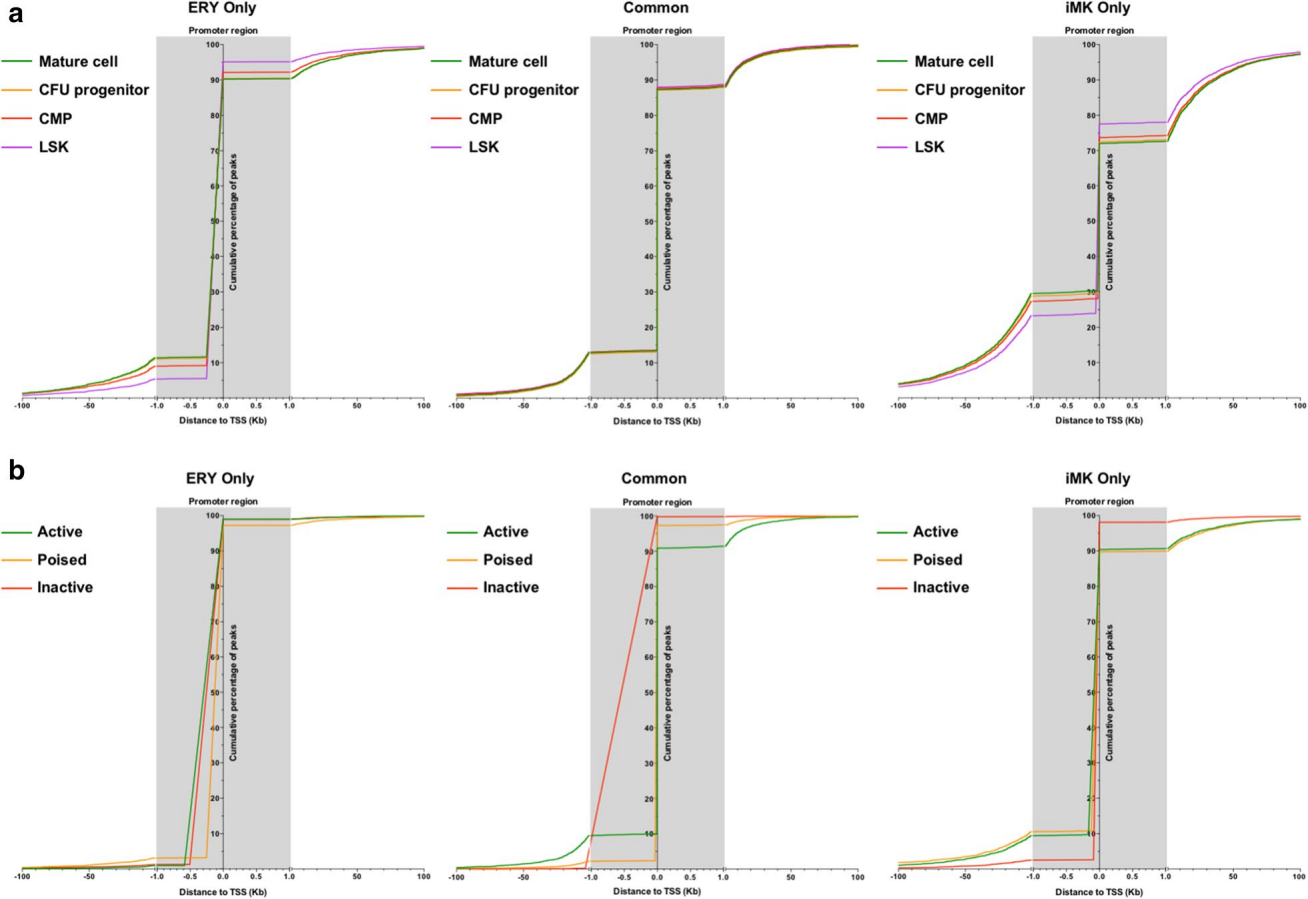
Distance of ARE to closest TSS. **a** Distance of LSK-established ARE to the transcriptional start site. **b** Distance of active, poised, and inactive ARE to the transcriptional start site

We plotted the proximity of AREs established during hematopoiesis to the closest TSS. AREs within 1 Kb of the TSS were defined as candidate promoter elements (cPE), and AREs outside of this region were defined as candidate enhancer elements (cEE). Based on these criteria, approximately 85% (1800) of ERY-specific and 55% (3500) of iMK-specific AREs established during differentiation were categorized as cPE (Fig. 7a), with primitive cells having more cPE than committed cells. We also observed that cEE established de novo during differentiation tended to form closer to the TSS in both ERY and iMK populations. Together these data demonstrate that ERY-specific AREs established early in hematopoiesis are more likely to be cPE, whereas a substantially larger fraction of iMK-specific AREs are comparatively more likely to be cEE.

In addition to AREs established at different stages of hematopoiesis, we plotted the proximity of active, poised, and inactive AREs to the nearest TSS. As with ERY-specific AREs established during differentiation, ERY-specific active, poised, and inactive AREs were almost exclusively categorized as cPE (Fig. 7b). While iMK-specific inactive AREs are almost exclusively defined as cPE, approximately 20% of active and poised AREs were classified as cEE. We conclude that LSK-established ERY-specific AREs are more likely to be candidate promoters, whereas a larger proportion of LSK-established iMK-specific AREs qualify as candidate enhancers.

AREs often contain consensus sequences for transcription factor binding sites that regulate cellular development and lineage specificity. We identified instances of consensus transcription factor binding site motifs (TFBS motifs) that were significantly enriched (*q*-value ≤ 0.05) in the set of AREs (Table 4, Additional file 3: Table S1). Poised iMK-specific AREs included motifs for ETS-family proteins FLI1 (*q*-value 0.029), ETS1 (*q*-value 0.004), and PU.1 (*q*-value 0.004). In addition to those found in poised iMK-specific AREs, active iMK-specific AREs also had matches to motifs for RUNX1 (*q*-value 0.046) and RUNX2 (*q*-value 0.010). ERY-specific AREs established in CMP were enriched for TFBS motifs of the master regulators GATA1, GATA2, and NF-E2 (*q*-value ≤ 0.03) (Table 4). Note that while many TFBS motifs are enriched in AREs present at all stages of hematopoiesis, Table 4 only shows those motifs whose enrichment passes a stringent FDR threshold of *q*-value ≤ 0.05. In summary, our data suggest that megakaryocytes maintain epigenetic and transcriptional profiles present in the progenitor cell populations, while erythroblasts acquire their distinct profiles later in hematopoiesis.

**Table 4:** Partial list of TFBS motifs enriched in AREs at different stages of hematopoiesis

Transcription factor	ERY-specific AREs				MEG-specific AREs			
	LSK (active)	LSK (poised)	CMP	CFU-E	LSK (active)	LSK (poised)	CMP	CFU-MK
AP-1						0.005	< 0.0001	< 0.0001
BACH1				0.001				
BATF						0.005	0.0001	0.002
CEBP							0.001	0.001
CHOP							0.0001	0.001
NF-E2			0.02	0.0002				
EHF					0.0001	0.005	< 0.0001	< 0.0001
ELF1					0.0001		< 0.0001	< 0.0001
ERG					0.0009		< 0.0001	< 0.0001
ETS					0.0001	0.0003	< 0.0001	< 0.0001
ETS1					0.0008		< 0.0001	< 0.0001
PU.1					0.0148	0.0039	< 0.0001	< 0.0001
SpiB					0.0004	0.0017	< 0.0001	< 0.0001
FLI1							< 0.0001	< 0.0001
IRF1					0.0138	0.0017	< 0.0001	< 0.0001
IRF2					0.0052		0.0001	0.0005
IRF8					0.0459			
RUNX1							0.0004	0.002
RUNX2							0.002	0.01
GATA1			0.03	0.01				
GATA2			0.03	0.01				
KLF3						0.0281		
SP1							0.04	

Benjamini-adjusted *q*-value for the enrichment score is reported

### The megakaryocytic lineage is associated with de novo DNA methylation

**Fig. 8:**
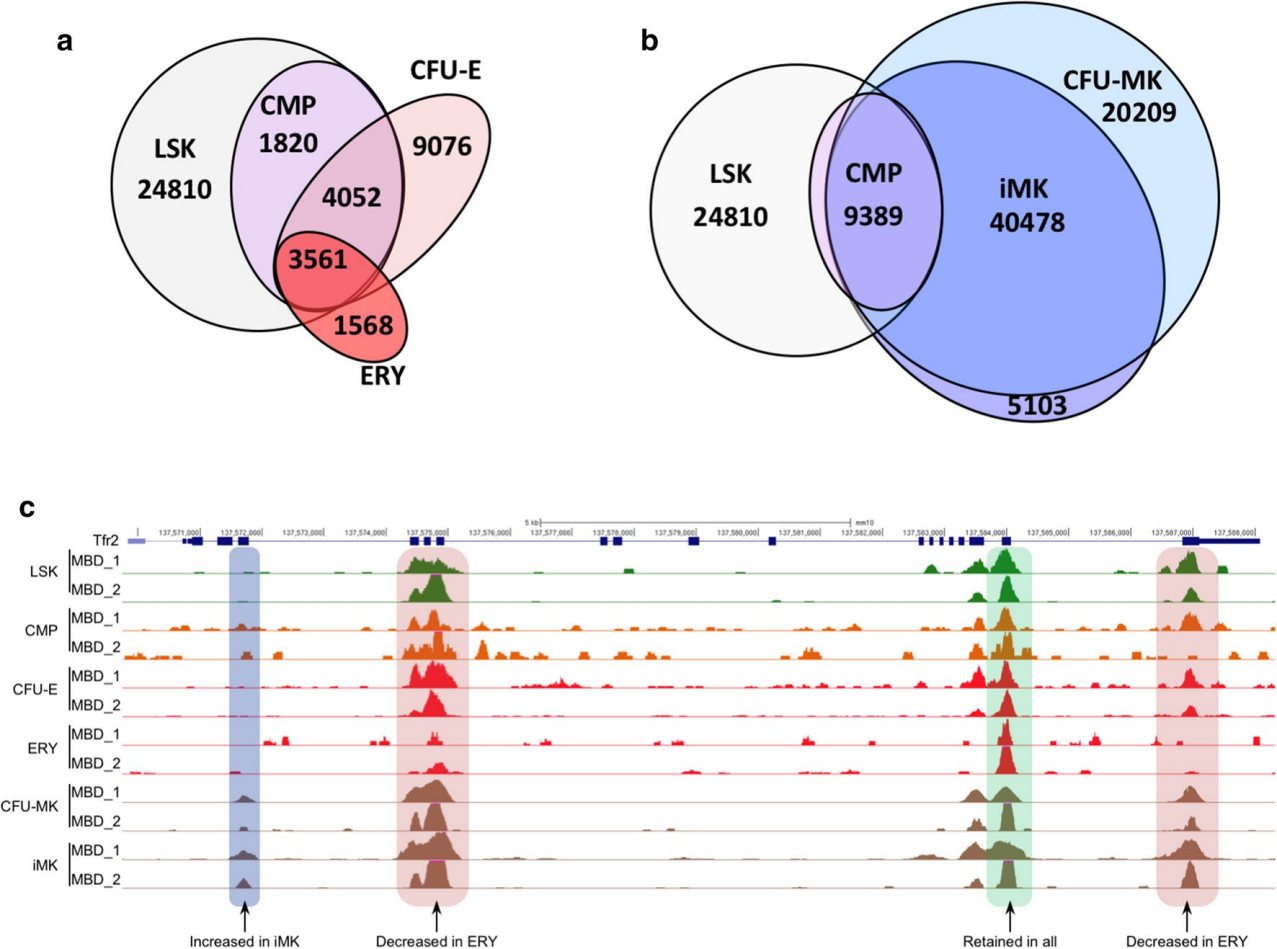
Overlap of DNA methylation and chromatin-accessible AREs. **a** Establishment of DNA methylation in the erythroid lineage. **b** Establishment of DNA methylation in the megakaryocytic lineage. **c** The Tfr2 locus showing example regions of maintained, lost, and de novo DNA methylation during erythropoiesis and megakaryopoiesis

We generated genome-wide DNA methylation maps in LSK, CMP, CFU-E and ERY, and CFU-MK and iMK populations using recombinant Methyl Binding Domain 2 (MBD2) protein to enrich methylated DNA fragments, followed by next-generation sequencing (MBD-Seq) [29]. We identified ~ 33,754 LSK and ~ 10,288 CMP methylation peaks; 86% of CMP peaks were present in LSK (Table 1). In differentiating erythroid cells, we observed a further loss of the DNA methylation peaks present in CMP accompanied by an additional ~ 9000 de novo methylation peaks in CFU-E. Mature erythroblasts had a global loss of methylation peaks accompanied by additional de novo methylation (Fig. 8a). Megakaryopoiesis was associated with an increase of ~ 60,000 de novo methylation peaks in CFU-MK, followed by a loss of ~ 20,000 of these de novo methylation peaks between CFU-MK and iMK (Fig. 8b). These patterns are illustrated in the transferrin receptor 2 (*Tfr2*) gene (Fig. 8c).

Because changes in DNA methylation are associated with differences in expression, we compared the overlap of DNA methylation peaks with cell-specific ARE profiles. We found that 43 (2.1%) of 2098 ERY-specific AREs established in ERY overlapped with ERY DNA methylation peaks (Table 5). 236 (11.5%) of 2054 ERY-specific AREs established in CFU-E overlapped with CFU-E DNA methylation peaks. In comparison, 789 (12.4%) of 6368 iMK-specific AREs established in iMK overlapped with iMK DNA methylation peaks, while 1121 (17.8%) of 6286 iMK-specific AREs established in CFU-MK overlapped with CFU-MK DNA methylation (Table 5).

**Table 5:** Comparison of DNA methylation and AREs established during hematopoiesis

ARE	DNA methylation			
	Mature cell	Committed progenitor	CMP	LSK
ERY-specific	43/2098 (2.1%) [ERY]	236/2054 (11.5%) [CFU-E]	64/1727 (3.7%)	207/1112 (18.6%)
iMK-specific	789/6386 (12.4%) [iMK]	1121/6286 (17.8%) [CFU-MK]	138/6068 (2.3%)	422/5385 (7.8%)

We compared the overlap of DNA methylation peaks with active, poised, and inactive regions in LSK. We found that nearly 20% of active and poised ERY-specific regulatory elements overlapped with regions of DNA methylation in LSK, whereas ~ 8% of active and poised iMK-specific regulatory elements overlapped with DNA methylation in LSK (Table 6). We conclude that, unlike the ATAC and transcriptional profiles, the methylation profile of the megakaryocytic lineage is acquired during differentiation while erythropoiesis is associated with global DNA demethylation.

**Table 6:** Comparison of DNA methylation and cell-specific regulatory elements in LSK

ARE	DNA methylation in LSK		
	Active	Poised	Inactive
ERY-specific	28/144 (19.4%)	122/605 (20.2%)	56/335 (16.7%)
iMK-specific	126/1651 (7.6%)	189/2489 (7.6%)	86/978 (8.8%)

## Discussion

It is well known that megakaryocytes and stem cells share a number of molecular features [10, 11]. Both HSC and megakaryocytes have similar dependencies on the thrombopoietin receptor MPL and express CD41 and CD117 (cKit) [10, 11, 30]. HSC and megakaryocytes also have similar dependencies on transcription factors, including expression of RUNX1, GATA2, and EVI1 [11]. Our data suggest that there is a large population of megakaryocyte-primed cells within the multipotent progenitor population (MPP) that shares the chromatin, enhancer/promoter, and transcriptional profiles of the MPP. It is also possible that only a subpopulation of HSC are megakaryocyte-primed, but our data suggest that the majority of MPP share transcriptional and regulatory characteristics with those of the megakaryocyte population [4, 15, 31–34].

The clearest distinction between MPP and megakaryocytes is in their DNA methylation profiles, where de novo methylation increases in CFU-MK but subsequently decreases in iMK [29]. Similar patterns have been observed in muscle and neuronal lineages (reviewed in [35–37]). Our data suggest that de novo methylation is an important step in megakaryopoiesis [38], while the chromatin accessibility, enhancer/promoter profiles, and transcriptional programs remain highly consistent. Changes in DNA methylation have been linked to induction of several megakaryocytic processes, including endomitosis, transcription factor expression and DNA affinity, and enhancer activity [39–42]. The requirement for de novo DNA methylation in megakaryopoiesis suggests that despite similar transcriptional and regulatory profiles with MPP, megakaryopoiesis does not constitute a “default” developmental program.

In contrast to megakaryopoiesis, erythropoiesis is associated with significant changes in the chromatin, enhancer/promoter, transcriptional, and methylation profiles. Our data suggest that, for most elements, these profiles are first detected at the level of the MEP, where the MEP ATAC-Seq profile clusters with that of mature erythroid cells. These data are consistent with the greater frequency of erythroid committed cells in the human MEP population [14]. Based on the transcriptional profiles, we propose that many cells in the mouse MEP population are in transition from multipotent progenitor cells (as evidenced by the similar transcriptional profiles) to erythroid committed cells. Our observations support this model and previous publications: ERY-specific AREs in CMP, but not LSK, contain TFBS associated with GATA-switching. However, in-depth single-cell analyses will be needed to test this hypothesis [43, 44].

The classic model of hematopoiesis is displayed as a hierarchy in which multipotent progenitors traverse a series of oligopotent and bipotent intermediates that progressively restrict lineage potential [1, 45, 46]. Recent studies, including single-cell transcriptional analyses and clonogenic assays, have identified a subset of megakaryocyte-primed progenitor cells in the MPP compartment that is proposed to give rise directly to megakaryocytes [4, 12, 15, 47, 48]. Our data are consistent with this model. Further down the hierarchy, multipotent progenitors give rise to common myeloid progenitor (CMP) cells, then to the megakaryocyte-erythroid lineage at the MEP stage, and finally to either the megakaryocyte or erythroid lineages [9, 49]. The existence of many of the cell types in this hierarchy is supported by the ability to grow colonies comprised of single or multiple lineages from single cells in semisolid medium in vitro. However, there are no reliable assays to culture both erythroid and megakaryocytic cells under the same conditions. Our data support models in which many cells within the classically defined MEP compartment have already become committed to one or the other lineage.

The data and analysis in this paper were generated as part of ValIdated Systematic IntegratiON of hematopoietic epigenomes (VISION) project, which aims to generate comprehensive catalogs of genomic regulatory elements in mouse and human hematopoietic cells and to conduct integrative statistical modeling and machine learning to predict regulatory interactions that are then validated by manipulating hematopoiesis at the genetic level. The data come both from efforts in the project laboratories, such as those generated for this paper, and also from other laboratories and consortia such as the Encyclopedia of DNA Elements (ENCODE) project and the NIH Roadmap Epigenomics Project [21–24]. These data are available at our website, usevision.org, for use by the larger community.

## Conclusion

Our studies confirm that the majority of the epigenetic and transcriptional profiles found in the hematopoietic stem cell population are present in megakaryocytes, while the erythropoietic epigenetic and transcriptional programs are not established until erythroid-lineage commitment. By performing “bulk” analyses, we achieve greater depth of coverage that emphasizes the dominant characteristics of cell populations. In contrast, single-cell analyses can resolve finer distinctions between populations, but at a lower depth of coverage.

## Methods

### Cell isolation

All primary hematopoietic cell populations were enriched from 5-to-8-week-old C57BL6 male mice. LSK, CMP, MEP, GMP, CFU-E, ERY, CFU-MK, and iMK populations were collected from bone marrow. Following collection, samples were lineage-depleted using antibodies (Fig. 1) and conjugated to BioMag Goat anti-Rat IgG magnetic beads (cat # 310007, Qiagen, Venlo, Netherlands). Lineage-depleted cells were then stained with fluorescently conjugated antibodies and sorted by flow cytometry on an FACSAria II (BD Biosciences). Neutrophils (NEU) and monocytes (MONO) were isolated directly from peripheral blood (PB). After Ficoll separation NEU were collected from the cell pellet following red cell lysis and MONO (Gr-1+Mac-1+ were sorted from the mononuclear fraction. All antibodies are listed in Table 7.

**Table 7:** List of antibodies

Antibody	Clone	Catalog number
Rat α-CD4	clone GK1.5	14-0041-86
Rat α-IL7Ra	clone A7R34	14-1271-85
Rat α-CD8	clone 53-6.7	14-0081-86
Rat α-Mac-1	clone M1/70	14-0112-86
Rat α-Gr-1	clone RB6-8C5	14-5931-86
Rat α-B220	clone RA3-6B2	14-0452-86
Rat α-Ter119	clone TER-119	14-5921-85
Rat α-Sca-1 (PE)	clone D7	12-5981-83
Rat α-CD34 (FITC)	clone RAM34	11-0341-85
Rat α-CD16/32 (PeCy7)	clone 93	25-0161-82
Rat IgG2b	clone eB149/10H5	14-4031-85
Rat IgG1	eBRG1	14-4301-85
Rat IgG2a serum	eBR2a	14-4321-85
Rat α-cKit (APC)	clone 2B8	17-1171-83
Rat α-CD41 (PE)	clone eBioMWReg30	12-0411-83
Rat α-CD61 (FITC)	clone 2C9.G3	11-0611-82
Rat α-Sca-1 (PerCP-Cy5.5)	clone D7	45-5981-82
Rat α-Ter119 (APC-780)	clone TER-119	47-5921-82
Rat α-CD44 (eFluor450)	clone IM7	48-0441-82
Rabbit a-H3K27ac	polyclonal	ab4729
Rabbit a-H3K4me1	polyclonal	ab8895
Rat α-Mac-1 (eFluor450)	clone M1/70	48-0112-82
Rat α-Gr-1	clone RB6-8C6	45-5931-80

All antibodies were purchased from eBiosciences (San Diego, CA, USA)

### RNA-Seq

Cells were sorted into media and total RNA was extracted using TRIzol and the Ambion PureLink RNA Mini Kit (Life Technologies, cat# 12183018), treated with DNase to remove genomic DNA using the DNA-free Kit (Life Technologies, cat# AM1906). Sequencing libraries were then constructed from 100 ng of treated, total RNA using the ScriptSeq Complete Kit (Illumina cat# BHMR1224) according to manufacturer’s specifications. In brief, the RNA was subjected to rRNA depletion using the Ribo-Zero removal reagents and fragmented. First-strand cDNA was then synthesized using a 5’ tagged random hexamer and reverse transcription, followed by annealing of a 5’ tagged, 3’-end blocked terminal-tagged oligo and second-strand synthesis. The Di-tagged cDNA fragments were purified, barcoded, and PCR-amplified for 15 cycles.

The size and quality of each library were then evaluated by Bioanalyzer 2100 (Agilent Technologies, Santa Clara, CA), and quantified using qPCR. Libraries were sequenced in paired-end mode on the NextSeq500 to generate 2 × 75 bp reads using Illumina-supplied kits as appropriate. The sequence reads were processed using the ENCODE3 long RNA-Seq pipeline (https://www.encodeproject.org/pipelines/ENCPL002LPE/). In brief, reads were mapped to the mouse genome (mm10 assembly) using STAR v2.5.4 [50], followed by RSEM v1.3.0 [51] for gene quantifications. RNA-Seq was repeated to generate at least two biological replicates. Analyzed data are included as Additional file 4.

### ATAC-Seq

Approximately 50,000 cells were collected by centrifugation at 600 × g for 10 min at 4 °C. Cells were washed once with cold 1× PBS and centrifuged as above. Cells were lysed by gently pipetting to resuspend cell pellet in cold lysis buffer (10 mM Tris–HCl pH 7.4, 10 mM NaCl, 3 mM MgCl2, 0.1% IGEPAL CA-630) and centrifuged as above. For each transposition reaction, cells were suspended in the following mix: 25 µl 2X Tagment DNA buffer (Illumina cat# FC-121-1030), 3 µl Tn5 Transposase (Illumina cat#FC-121-1030), 22 µl nuclease-free H2O and incubated for 30 min at 37 °C. Reactions were terminated by adding 5 µl of 1% SDS solution and purified using SPRIselect beads (Beckman Coulter cat #B23318) at a 1:1 ratio according to manufacturer’s instructions, followed by a right side size selection using SPRIselect beads (Beckman Coulter cat #B23318) at a 0:5 ratio according to manufacturer’s instructions. Following purification, library fragments were amplified using 1 × NEBnext PCR master mix and 1.25 μM of custom Nextera PCR primers 1 and 2 [52] using the following PCR conditions: 72 °C for 5 min; 98 °C for 30 s; and thermocycling at 98 °C for 10 s, 63 °C for 30 s, and 72 °C for 1 min. To reduce GC and size bias, the PCR was monitored using qPCR in order to stop amplification before saturation. To this end, libraries were amplified for five cycles, and then a 5 µl aliquot of the PCR was removed and added 10 µl of the PCR cocktail with SYBR Green at a final concentration of 0.6×. This reaction was amplified for 20 cycles to determine the additional number of cycles needed for the remaining 45-μL reaction. Libraries were amplified for a total of 17–19 cycles and then purified using AMPure XP beads (Beckman Coulter cat #A63881) at a 1:1 ratio according to manufacturer’s instructions. Constructed libraries were run on the Agilent Bioanalyzer 2100 (Agilent Technologies) using the 7500 DNA kit (cat# 5067-1504) as appropriate to determine the average size and confirm the absence of unligated adaptors. Libraries were quantitated by qPCR using the Kapa SYBR FAST Universal kit (Kapa Biosystems) according to the Illumina’s Sequencing Library qPCR Quantification Guide. Libraries were multiplexed and sequenced on the Illumina NextSeq500 using Illumina’s kits and reagents as appropriate. ATAC-Seq was repeated to generate at least two biological replicates. Reads were mapped using Bowtie v1.0.0 [53], accessible regions identified with FSeq v1.85 [54], and peaks called using HOMER v1.0 [55]. To identify consensus peak sets, peaks from all datasets were combined and merged. Peaks present in both replicates within the same merged region were used for further analysis. Analyzed data are included as Additional file 5.

### ChIP-Seq

Approximately 20 × 10^6^ cells were fixed in 0.4% formaldehyde (16% methanol-free, Thermo Scientific) for 15 min before quenching in 125 mM glycine for 5 min. Cells were washed in 2X PIC (Roche mini-tabs, 1 tab in 5 ml = 2X) and stored at − 80°C. Cells were resuspended in lysis buffer (10 mM Tris–HCl, pH8.0, 10 mM NaCl, 0.2% NP40) and sonicated (QSonica) to a size range of 200–500 bp as determined by electrophoresis. Immediately prior to immunoprecipitation, an aliquot was removed to be sequenced as a matched input control. Immunoprecipitation was performed using H3K27ac (Abcam ab4729) supplemented with 50 mM sodium butyrate. DNA fragments were purified with AMPure XP beads (cat # A63880, Agencourt, Pasadena, CA, USA) according to the manufacturer’s protocol. Sequencing was performed at the NIH Intramural Sequencing Center on an Illumina HiSeq2500. Chromatin immunoprecipitations were repeated to generate four biological replicates. Sequence data were aligned to the mm10 genome using Bowtie v2.3.4 [56] and peak calling performed using SICER v1.1 [57]. Peaks present in all replicates were used for further analysis, as above.

We accessed indexing-first immunoprecipitation (iChIP) experimental data performed by Lara-Astiaso et al. [27] from Gene Omnibus (GSE60103). Sequencing reads from long- and short-term HSC (LT-HSC and ST-HSC, respectively) and multipotent progenitor cells were combined to generate “LSK” profiles from the iChIP data. These reads were aligned using Bowtie v2.3.4 and peak calling performed using MACS2 [58]. We combined LT-HSC, ST-HSC, and MPP peak sets to create the iChIP “LSK” population for downstream comparisons. For studies involving indexing-first chromatin immunoprecipitation data (iChIP), reads were accessed via GSE60103 (Lara-Astiaso et al. [27]), data aligned using Bowtie v2.3.4 and peaks-calling performed using MACS2 [58]. To create a set of LSK data, peaks were combined from the long-term HSCs, short-term HSCs, and multi-potential progenitor sample sets. Analyzed data are included as Additional file 6.

### MBD-Seq

Genomic DNA was isolated from enriched cells with the QIAGEN Puregene kit and sonicated to 200- to 400-bp fragments (QSonica). MBD2 enrichment was performed with the Active Motif Methyl Collector kit. Approximately 1 μg of sonicated genomic DNA was incubated with MBD2-His-conjugated protein and magnetic beads according to the manufacturer’s protocol. After enrichment, both the methylated fraction and supernatant fractions were purified with QIAGEN DNA purification columns. Quantitative PCR amplification of the differentially methylated regions regulating the imprinting of Snrpn and Rasgrf1 and the unmethylated CpG island promoter of Actb was performed with SYBR Green PCR master mix (Applied Biosystems) and was used to validate the enrichment of methylated DNA using the MBD2-pull-down approach.

Two biological replicates of each enriched cell population and one supernatant sample per cell type were submitted for high-throughput sequencing analysis. Between 225 and 540 ng of MBD2-enriched DNA and 1 μg of supernatant for each cell type were used to construct DNA libraries according to the Illumina protocol. The libraries were sequenced on the Illumina Genome Analyzer platform, and 36-bp single-end reads were used to uniquely identify the MBD2-bound fraction of the mouse genome. Sequenced reads were mapped to the mouse genome (UCSC assembly mm10) using Bowtie v2.3.4 [56]. Peaks for each cell type were called using the SigSeeker peak calling ensemble [59]. Reads from the matched supernatant were used as a control for each cell type. Replicate peaks that were called by two or more peak calling tools and overlapped by at least 100 bp were considered for further analysis. Analyzed data are included as Additional file 6.

### Additional data analysis and visualization

Permutation tests were performed using the R package regioneR v1.10.0. Transcription factor enrichment analyses were performed in HOMER v3.18 [55]. Graphs were created in PRISM v6.

## Additional files

**Additional file 1: Fig. S1.** Establishment of ERY and iMK enhancer/promoter regions throughout hematopoiesis. (A–C) LSK-accessible cell-specific and shared AREs were compared against the indexing-first H3K4me1 and H3K27ac chromatin immunoprecipitation profiles in (A) LT_HSC, (B) ST_HSC, and (C) MPP (Lara-Astiaso et al., Science, 2014). (D) Z-score of overlap between ARE and iChIP peaks. Hashed bars indicate p-value >0.05.

**Additional file 2: Fig. S2.** Locus-specific example of (epi) genomic correlations. (A) Erythroid specific induction of AREs and expression (*Slc4a1*) and constitutive AREs and expression (*Slc25a39*). RNA-Seq and ATAC-Seq are shown for all 10 cell types, and histone modifications for 5 cell types of most relevance. (B) Retention of AREs and expression from LSKs in MKs and loss in ERY. RNA-Seq and ATAC-Seq are shown for all 10 cell types, and histone modifications for 5 cell types of most relevance. Tracks are displayed on the mm10 genome.

**Additional file 3.** Enrichment scores of transcription factor binding sites in AREs. Unabridged list of enriched transcription factor binding sites in AREs. Scores are reported as q-values.

**Additional file 4.** RNA-Seq data with TPM calculations. Transcripts per million counts are included for each RNA-Seq replicate.

**Additional file 5.** Peak data from ATAC-Seq experiments. The presence of ATAC-Seq peaks in each cell type is indicated by a 1 (present) or 0 (not present).

**Additional file 6.** Peak data from ChIP-Seq and MBD-Seq experiments. Peaks from H3K27ac ChIP-Seq and MBD-Seq are presented in BED format. Note that due to file restrictions, MBD-Seq data for CFU-MK is included across two workbooks.

## Abbreviations

HSC: hematopoietic stem cell
LSK: Lin^−^Sca^+^Kit^+^
MPP: multipotent progenitor
CMP: common myeloid progenitor
CLP: common lymphoid progenitor
GMP: granulocyte/macrophage progenitor
MEP: megakaryocyte/erythroid progenitor
CFU-MK: colony-forming unit (megakaryocyte)
CFU-E: colony forming unit (erythrocyte)
ERY: erythrocyte
MK: megakaryocyte
VISION: ValIdated Systematic IntegratiON of hematopoietic epigenomes
ENCODE: Encyclopedia of DNA Elements
iMK: immature megakaryocyte
ATAC-Seq: assay for transposon-accessible chromatin followed by next-generation sequencing
RNA-Seq: next-generation sequencing of RNA
ChIP-Seq: chromatin immunoprecipitation followed by next-generation sequencing
MBD-Seq: methyl-binding protein immunoprecipitation followed by next-generation sequencing
TPM: transcripts per million count
PCA: principle component analysis
ARE: active regulatory element
iChIP: indexing-first chromatin immunoprecipitation
TSS: transcriptional start site
cEE: candidate enhancer element
cPE: candidate promoter element
TFBS: transcription factor binding site
