# Supplementary figures and images for: Establishment of regulatory elements during erythro-megakaryopoiesis identifies hematopoietic lineage-commitment points
Source: Epigenetics Chromatin. 2018 May 28;11:22. doi: 10.1186/s13072-018-0195-z (PMC5971425; doi:10.1186/s13072-018-0195-z)

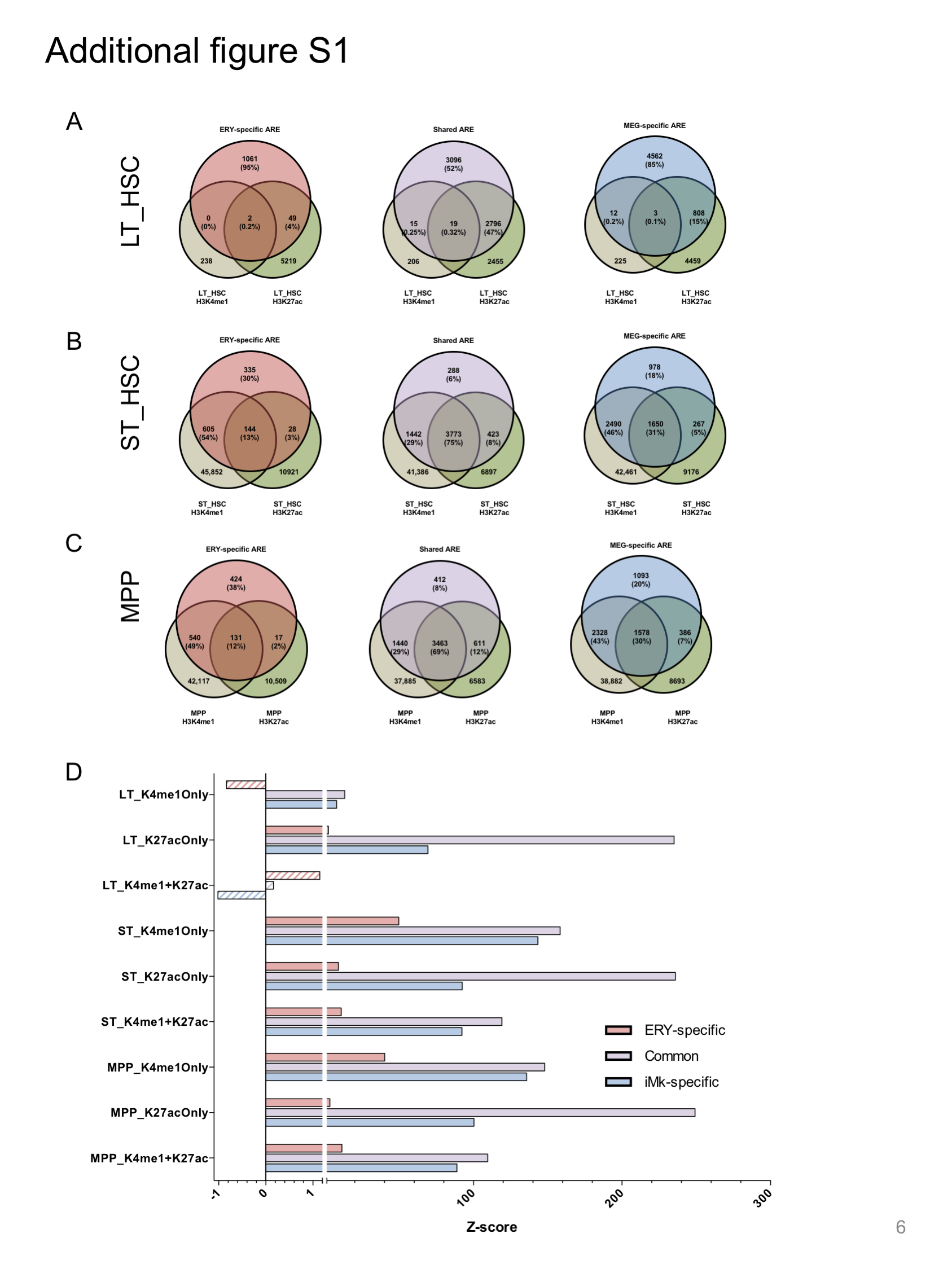

Supplement: Supplementary file 1 — Additional file 1: Fig. S1. Establishment of ERY and iMK enhancer/promoter regions throughout hematopoiesis. (A–C) LSK-accessible cell-specific and shared AREs were compared against the indexing-first H3K4me1 and H3K27ac chromatin immunoprecipitation profiles in (A) LT_HSC, (B) ST_HSC, and (C) MPP (Lara-Astiaso et al., Science, 2014). (D) Z-score of overlap between ARE and iChIP peaks. Hashed bars indicate p-value >0.05. [file 13072_2018_195_MOESM1_ESM.png]

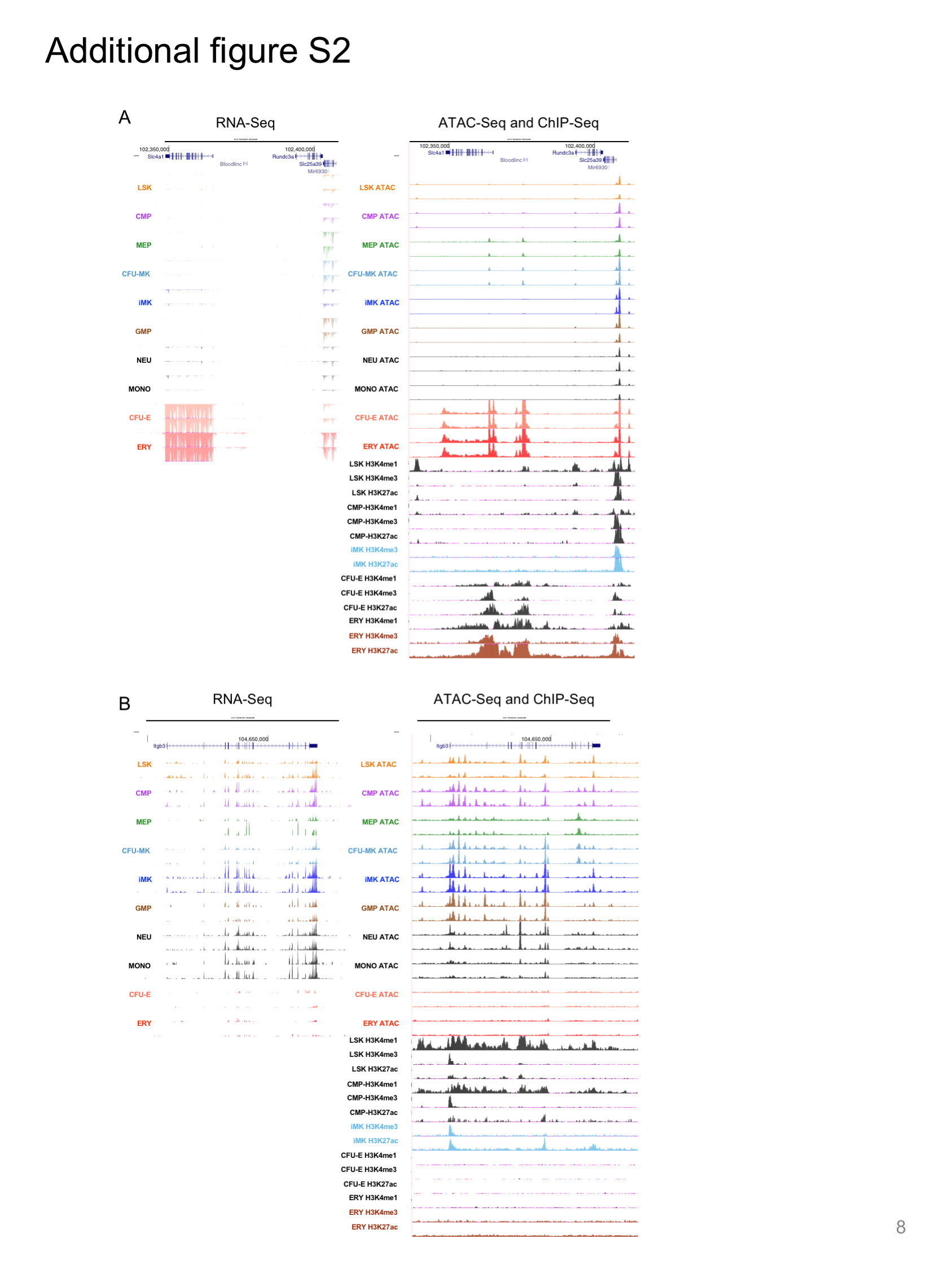

Supplement: Supplementary file 2 — Additional file 2: Fig. S2. Locus-specific example of (epi) genomic correlations. (A) Erythroid specific induction of AREs and expression (Slc4a1) and constitutive AREs and expression (Slc25a39). RNA-Seq and ATAC-Seq are shown for all 10 cell types, and histone modifications for 5 cell types of most relevance. (B) Retention of AREs and expression from LSKs in MKs and loss in ERY. RNA-Seq and ATAC-Seq are shown for all 10 cell types, and histone modifications for 5 cell types of most relevance. Tracks are displayed on the mm10 genome. [file 13072_2018_195_MOESM2_ESM.png]
